# Sequestration of CO_2_ by halotolerant algae

**DOI:** 10.1186/2052-336X-12-81

**Published:** 2014-05-06

**Authors:** Udhaya Ramkrishnan, Benedict Bruno, Sandhya Swaminathan

**Affiliations:** 1 National Environmental Engineering Research Institute, CSIR-Complex, Chennai 600 113, India

**Keywords:** Halotolerant algae, CO_2_ sequestration, Biomass, Salinity

## Abstract

The potential of halotolerant algae isolated from natural resources was used to study CO_2_ fixation and algal lipid production. Biological fixation of CO_2_ in photobioreactor in presence of salinity is exploited. The CO_2_ concentration 1060 ppm gave the highest biomass yield (700 mg dry wt/l), the highest total lipid content (10.33%) with 80% of CO_2_ removal.

## Background

The world population has been growing rapidly and has nearly doubled in the last fifty years. This rapid growth has been accompanied by economic development both of which have resulted in high energy demand. Fossil fuels, coal oil and gas have been the major sources which have supplied this energy demand for a long time. However limited availability of these sources coupled with the adverse environmental impacts associated with their extraction and use have prompted the search of other renewable energy sources to meet the future energy demands. Several renewable energy sources such as solar, wind, hydel and biomass energy systems are in various stages of development and their applications are steadily increasing. However, one of the sources, which has attracted considerable attention in recent years is the biofuels such as bioethanol and biodiesel. Biofuels can play an essential part in reaching the target to replace petroleum based transportation fuels and in reducing CO_2_ emissions, in environmental and economic sustainability are considered carefully [1].

First generations of biofuels, which have attained economic levels of production, have been mainly extracted from food, oil crops and animal fats using conventional technology [2]. Second generation of biofuels have the potential to use waste residues and make use of waste land thereby promoting rural development and improve the economic conditions of developing countries.

The most promising second generation biofuel is biodiesel from algae which is capable of using CO_2_ and sunlight to produce a variety of organic molecules, particularly, carbohydrates and lipids. These photosynthetic organisms are known to produce high biomass yields with high oil content which can be cultivated in fresh water or wastewater [3]. Another advantage of algae is their ability to tolerate and adapt to a variety of environmental and nutritional conditions. The most positive impact is the utilization of atmospheric CO_2_ which can have a significant benefit in the context of global warming. However, the water demand for algae is as high as 11-13 million liters/ha/day for cultivation in open pond [4]. Their ability to grow in fresh water, municipal, industrial wastewaters and sea water not only overcomes this hurdle but also provides treated wastewater for other uses.

Unlike other sources of biofuels, algae have the capability to produce different types of biofuels. This multiproduct paradigm of algae makes it an ideal candidate for the concept of biorefining which involves production of many products from a raw material. The products produced from algal biomass are listed in Table 1. Considering the advantages of algae as a biofuel the present work investigated their effectiveness in CO_2_ sequestration. This paper discusses the effectiveness of a halotolerant algae for CO_2_ sequestration in a laboratory scale photoreactor with potential to produce biodiesel.

**Table 1:** Integrated production system of algal biomass: perspective products value and market

**Product**	**Example**	**Value**	**Market**
Fatty acids	Docosahexanoic acid	High	Food Ingredients
	Eicosapetanoic acid	Moderate	
	Alpha linolenic acid	Moderate	
	Arachidonic acid	High	
Carotenoids	Astaxantin	High	Food Ingredients
	Zeaxanthin	High	
	Lutein	High	
Fluorescent label	Phycoerythrin	High	Biomedical
	Phycocyanin		
Biofuel	Biodiesel	High	Transport industries
	Bioethanol		
	Biohydrogen		
Meal	Algal meal residual amount of docosohexanoic acid and eicosapentanoic acid	Moderate	Feeds
			(Poultry fish shrimp swine feed)
By product	Glycerin	Low	Biodiesel Industry, algal fuel
Chemicals	Algal alginin caragenin,1.3 Propanediol	Moderate	Biotechnology & Food industry
Nanotech devices	Silicon nanochips	High	Semiconductor, nanotech

## Methods

### Algal feedstock

The algal culture was isolated from an agricultural runoff using the medium described by Fiore et al. [5]. The medium has the following composition: (mM): MgSO_4_.7H_2_O, 162.3; CaCl_2_ 2H_2_O, 81.6; NaCl, 684.5; and microelements. The microelement stock containing (mM): H_3_BO_3_, 9.25; MnCl_2_ 4H_2_O,1.82; ZnSO_4_.7H_2_O,0.15; Na_2_MoO_4_.2H_2_O, 0.25; CuSO_4_.5H_2_O,0.06; COCl.6H_2_O.,0.03; NH_4_VO_3_,0.04 and FeEDA solution 160 ml. The final pH of the medium was 7.8. Cultures were routinely checked for purity by microscopic examination and plating. The pure culture of halotolerant algae was identified as *Chlorella* sp by 18 S rDNA techque.

### Experimental

**Figure 1:**
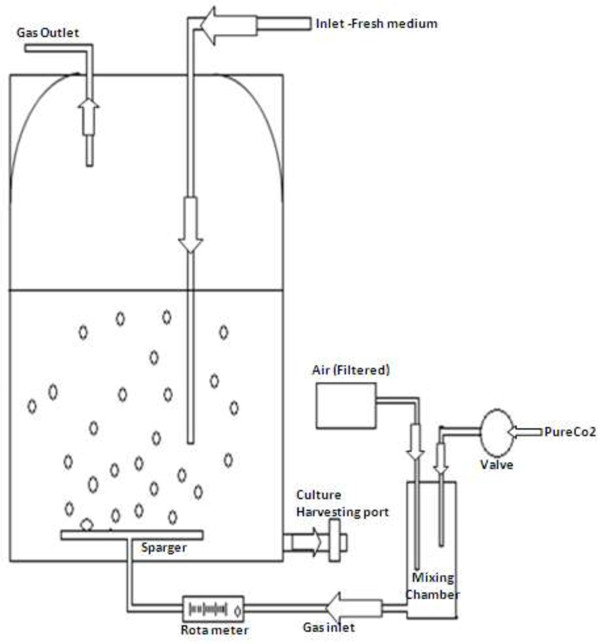
Schematic diagram of lab scale 2L Photo bioreactor.

Algal cultivation was done in photoreactors consisting of 2Lborosilicate glass bottles fitted with rubber stopper. CO_2_ from a gas cylinder was mixed with air to get desired concentration and bubbled through fine diffuser. The schematic of the experimental set up is shown in Figure 1. The flow rate (20 ml/min) of gas was measured using a rotameter. The photoreactor was irradiated using standard fluorescent lamps (40 w) placed on both the sides. The excess gas was discharged through an out let tube. The inlet and outlet gas samples were sampled at regular intervals and analyzed for CO_2_. The algal samples were collected from an outlet at regular intervals and analyzed for various parameters.

### Analytical methods

#### Algal biomass

The concentration of algal biomass was measured by measuring the optical density of the algal suspension at 680 nm wave length in a UV-visible spectrophotometer (Thermo Electron Corporation Type UV1, England). The dry weight of algae was estimated from a standard graph.

Alkalinity and pH of the suspension was measured. as per standard procedures [6].

#### Fatty acids estimation

Algal cells were harvested by centrifugation (10000 rpm) for 10 min. The cell pellets separated from the supernatant were washed with distilled water and dried. Fifty mg of dried algal biomass was taken in 15 ml of test tube, 1.6 ml of double distilled water, 4 ml methanol and 2 ml of chloroform were added and mixed thoroughly for 30 S. Thereafter, an additional 2 ml of chloroform and 2 ml of double distilled water were added and solution was mixed for 30 S. Following this, the mixture was centrifuged, at 5000 rpm for 10 min. The upper layer decanted and the lower chloroform layer containing the extracted lipids was collected in another test tube. The extraction procedure was repeated again with the residual pellet and both the chloroform extracts were mixed to gather and evaporated till dryness. The dried total lipids were measured gravimetrically and lipid content was calculated as percentage of algal biomass.

#### DNA isolation, PCR amplification

During CO_2_ sequestration, algae samples were processed for DNA extraction as method. 18S rDNA gene was amplified using universal eukaryotic primers F5′-GTCAGAGGTGAAATTCTTGGATTTA-3 and R 5′-AAGGGCAGGGACGTAATCAACG-3′ [7]. The PCR conditions were 30 cycles of denaturation at 95°C for 2 min. followed by annealing at 55°C for 2 min. and final extension at 72°C for 10 min. The reaction mixture content 5 ul DNA template, 1X PCR buffer and 5U Taq DNA polymerase to a final volume of 50 ul. The amplified product was resolved on 1.2% (w/v) agarose.

## Results and discussion

Since the objective of the study was to evaluate the CO_2_ sequestration potential of the isolated halotolerant algae the growth profile was measured at different CO_2_ concentrations.

### Growth of halotorerent algae

**Figure 2:**
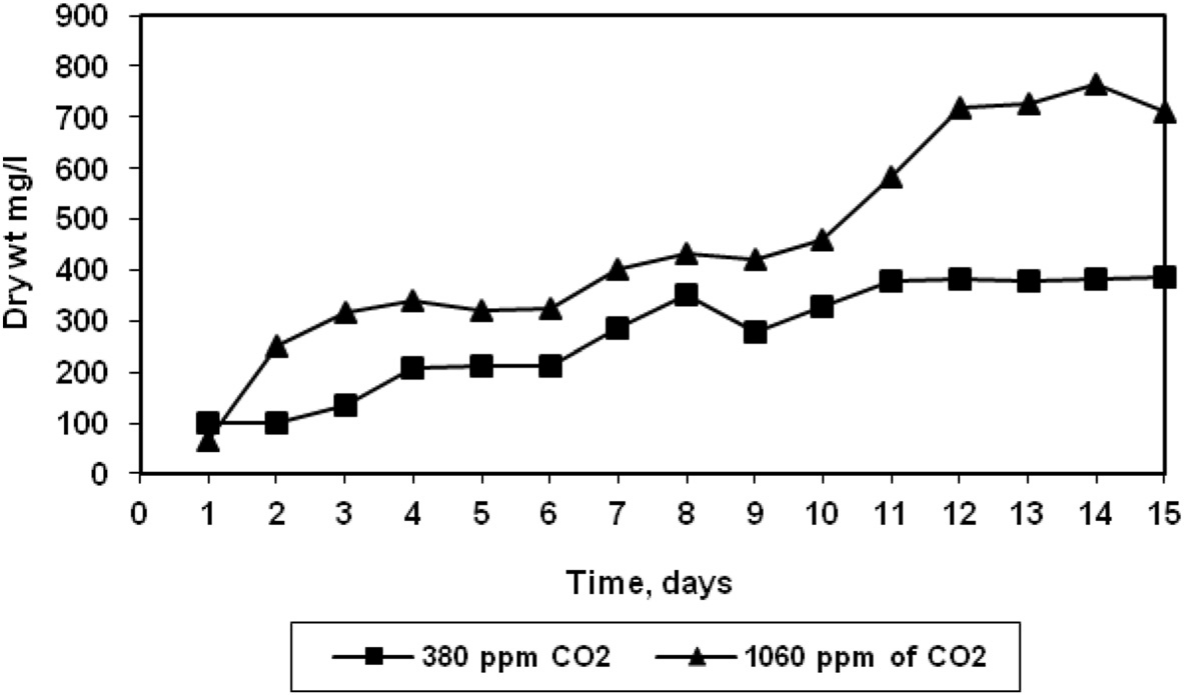
**Algal growths in presence of CO**_**2**_**.**

**Figure 3:**
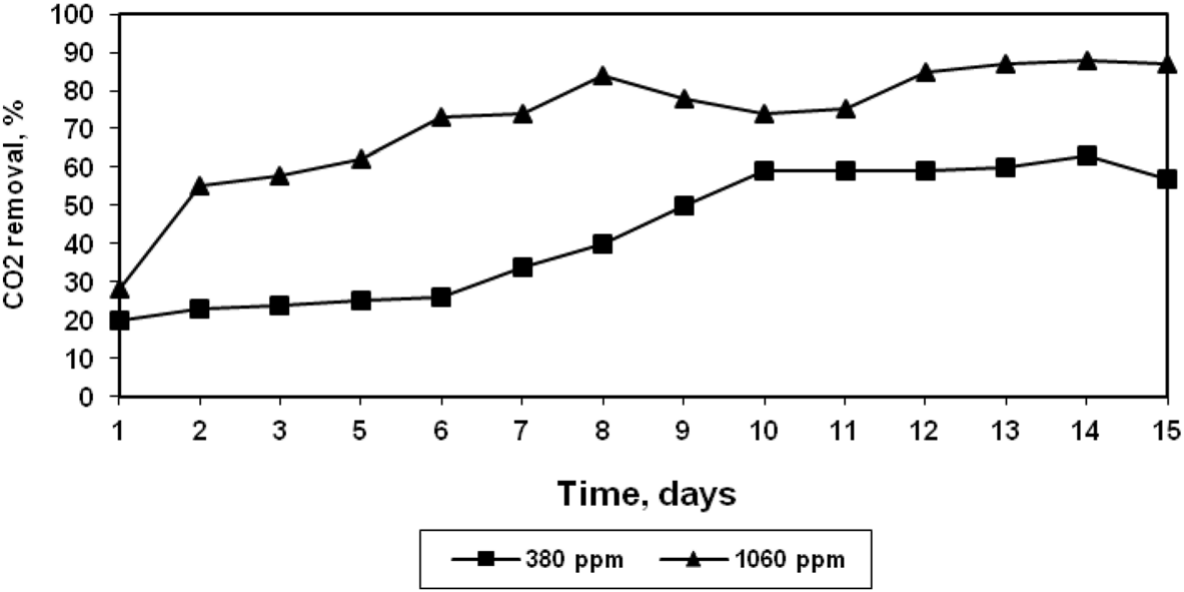
**CO**_**2 **_**removals by halotolerant algae.**

The growth profile of the halotolerant algae at two different CO_2_ concentrations are shown in Figure 2. At both the concentrations, the growth increased steady with time till 11 days after which a sharp increase in growth was observed for high CO_2_ concentration (1060 ppm) and stationery stage was reached after 14 days. Similar increase in *Chlorella vulgaris* growth with increasing CO_2_ concentration has been reported by Zeng et al. [8]. The lower growth rate of algae at low CO_2_ concentration may be attributed to insufficient CO_2_ supply. This is further confirmed from the CO_2_ removal efficiency at both the CO_2_ concentrations shown in Figure 3. The halotolerant algae were observed to be more efficient at higher CO_2_ concentration. The maximum removal efficiency increased from 60% to 90% as CO_2_ concentration increased from 380 to 1060 ppm. It was also observed that the trends of CO_2_ removal and growth were similar. Weissman and Tillett reported that microalgae could convert up to 99% of CO_2_ in solution [9].

**Figure 4:**
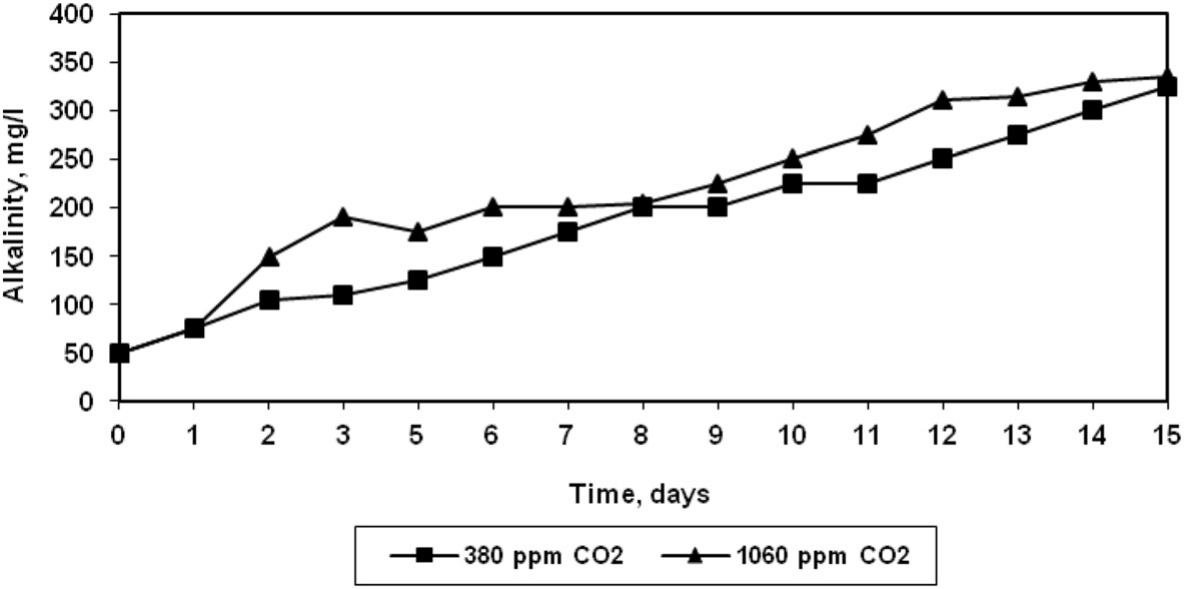
Variation in alkalinity during growth of halotolerant algae.

**Figure 5:**
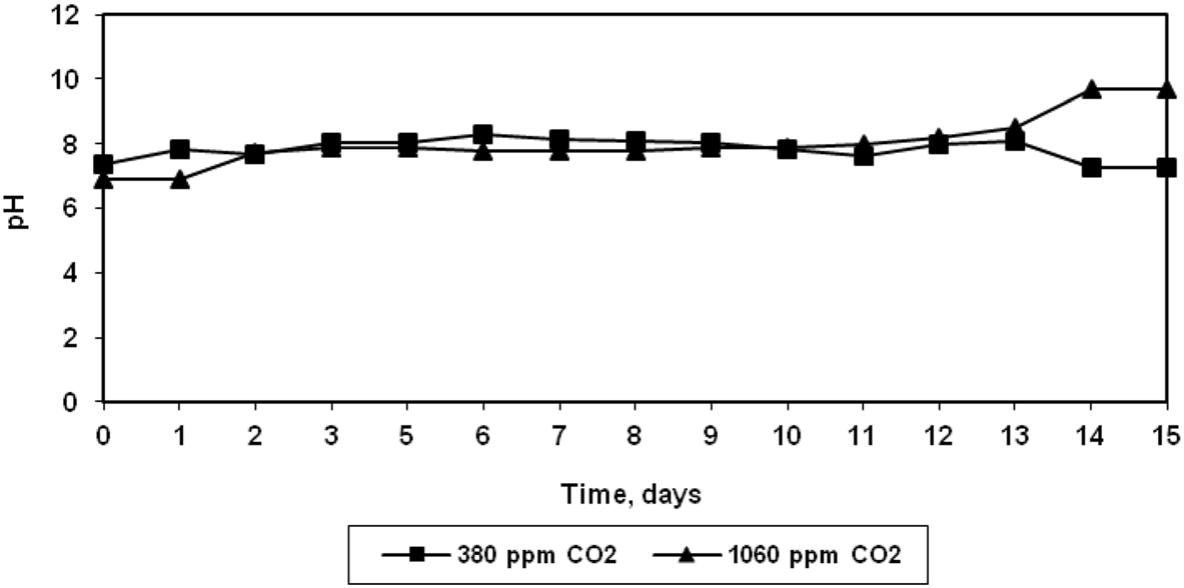
Variation in pH during growth of halotolerant algae.

Since CO_2_ is a weakly acidic gas, it affects the alkalinity of the solution. As seen from Figure 4, it was observed that the alkalinity increased from 60 to 350 mg/l during the growth of algae at both the CO_2_ concentrations. However, the pH of the solution remained constant throughout the growth at both CO_2_ concentrations (Figure 5). Rangarao et al. have observed that bubbling of CO_2_ continuously resulted in decrease in pH of culture solution thereby fall in cell density [10].

### Effect of salinity on growth of halotolerant algae

**Figure 6:**
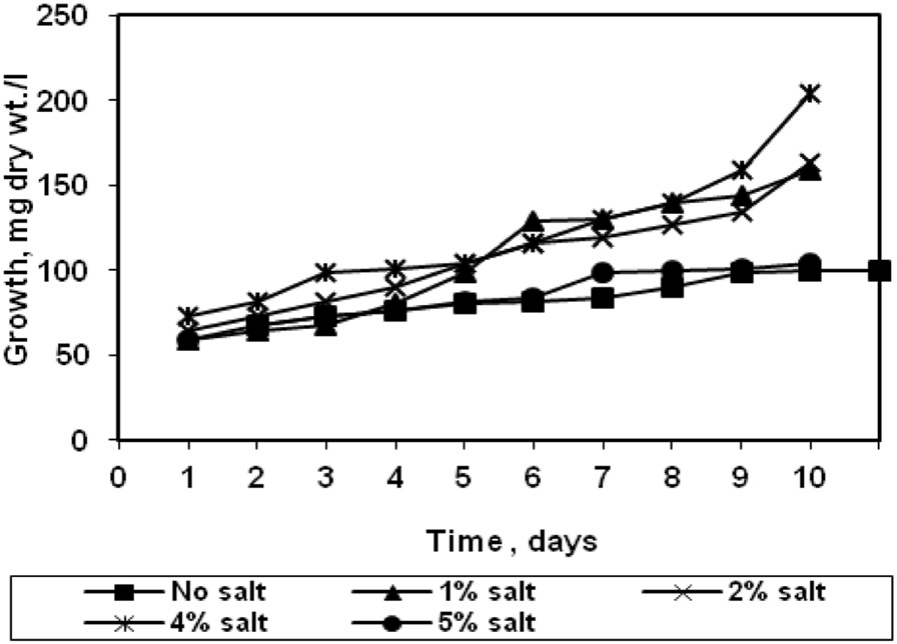
Effect of salinity on growth of halotolerant algae.

**Figure 7:**
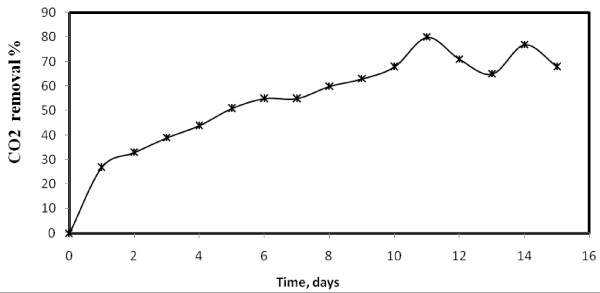
**CO**_**2 **_**removal by halotolerant algae at 4% salinity.**

Since the algae was isolated from agriculture runoff water containing relatively high salt concentration, the optimal salt concentration required for growth of algae was investigated. The result presented in Figure 6 show that the algal growth has increased with increased in salt concentration till 4% and it was relatively poor in absence. The removal of CO_2_ at 4% salt concentration is shown in Figure 7 and almost 80% of CO_2_ was removed till 11 days. This indicates the salt loving nature of the algae. However, when the salt concentration was increased above 4%, the growth rate decreased which indicates an optimal level for growth. Hence this species is termed as halotolerant algae. There is little impact on CO_2_ removal efficiency.

### Lipid content of halotolerant algae

The halotolerant alga was not only evaluated for its CO_2_ sequestration potential based on its growth, but also for its potential use as a feed stock for biodiesel. This was determined from the lipid content of the algal cell given in Table 2. It was observed that as the cell growth increased with the time, its lipid content was also increased, with a maximum of 10% in 14 days. However, this is much lower compared with the 20-40% lipid content reported for same algal strains [11]. Liu et al. reported that total lipid contents representing 20-50% of the dry weight were found to be quite common [12]. Go et al., reported 12.2 mg/g/day oil productivity in marine algae *Tetraselmis suecica*[13]. Higher salinity might be affecting lipid production pathway in algae.

**Table 2:** Effect of salinity on biomass, specific growth rate and lipid production from halotolerant algae

**Salt content**	**Biomass, mg/l**	**Specific growth rate, u/d**	**Fatty acid,%**
No salt	300	0.09	0.02
1%	304	0.08	0.03
2%	343	0.12	2.77
4%	711	0.17	10.33
5%	317	0.11	2.5

### Identification of halotolerant algae

**Figure 8:**
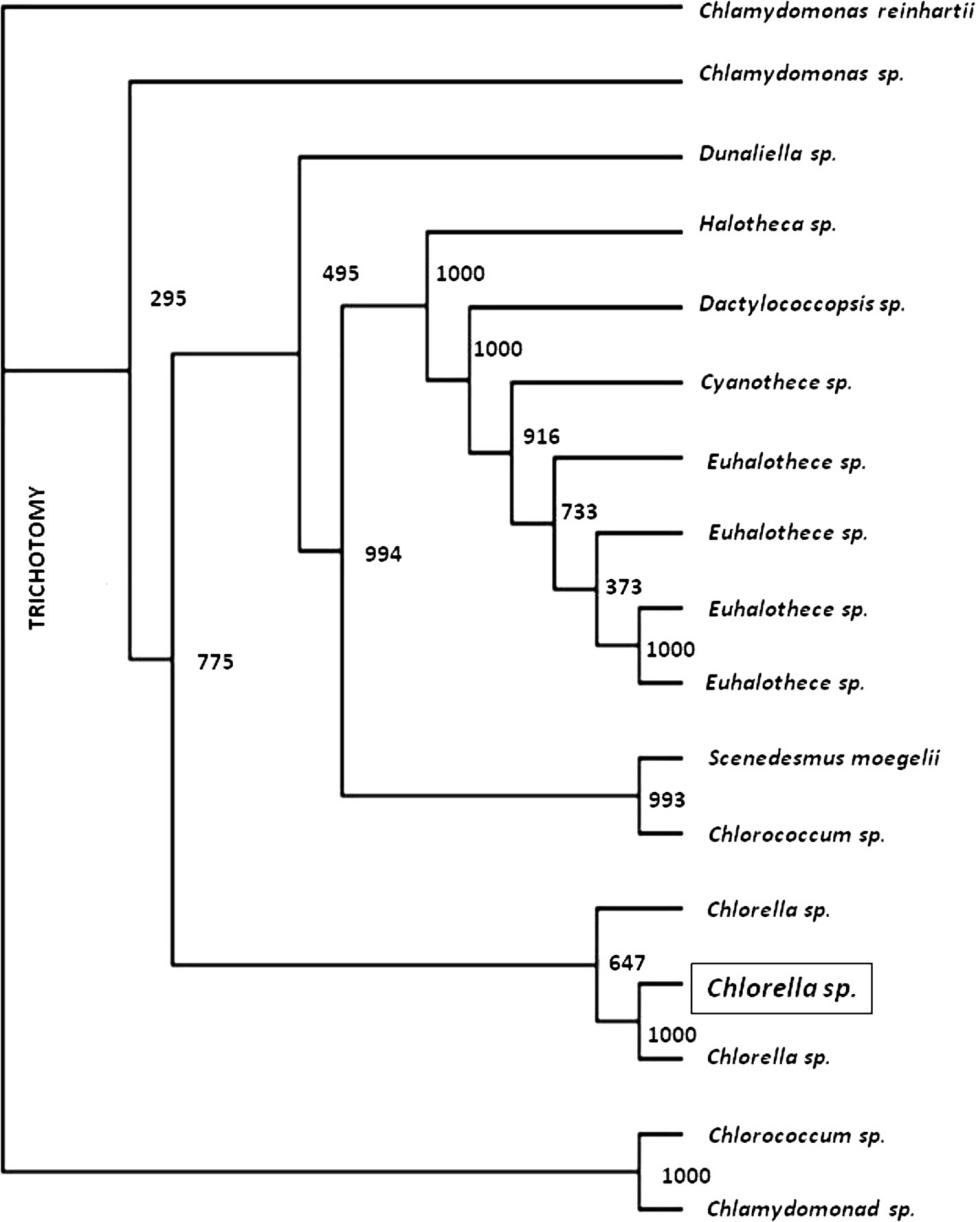
**Dendogram for algae.** KC492078 – Chlorococcum, KC492079 - Chlamydomonas sp., KC492080 - Chlorella sp., KC492081 - Chlamydomonas sp., KC218498 - Chlorella sp., KC166137 - Chlamydomonas reinhardtii, KC218488 - Scenedesmus maegelii, KC218482 - Chlorococcum sp., KC218500 - Dunaliella sp., JN934686 - Chlamydomonas sp., AJ000708 - Cyanothece sp., AJ000709 - Euhalothece sp., AJ000710 - Euhalothece sp., AJ000711 - Dactylococcopsis sp., AJ000712 - Euhalothece sp., AJ000713 - Euhalothece sp., AJ000724 - Halothece sp.

A phylogenic study based on 18S r DNA sequencing is one of the most useful methods for inferring relationship between genera or between the species belonging to a genus [14]. The identification of the halotorent strains isolated from the agricultural runoff was done based on DNA elongation using 18S rDNA primers in PCR and by comparison with 18S rDNA sequences of a library of species. Sixteen strains that were reported for CO_2_ sequestration were collected from NCBI and were used as reference strains for construction of a dendogram presented in Figure 8. They were identified following the BLAST analysis of the 18S rRNA gene sequence and based on >97% 18S rRNA gene sequence similarity, the nine isolates were categorized to two groups comprising of two sub groups. Based on the phylogeny, it was found that these reference strains mainly belonged to the phylum *Chlorophyceae*. From the nearest phylogenic neighbour of the reference strains showing 100% similarity, the halotolerant algae isolated in study identified as *Chlorella* species and its gene bank accession no KC492080. Among the thirteen strains, five strains belonged to the family C*hlamydomonadaeceae* and *Chlorococcaceae* showing close similarity (64.7%) with that of the neighbour *Chlorella* sp. (KC166137) that was reported to grow under heterotrophic condition.

## Conclusions

A halotolerant algal strain was isolated from agricultural runoff and its potential for CO_2_ sequestration was evaluated. The strain was found to grow well at a salt concentration of 4% and yielded 204 mg/l biomass in 14 days. The cell growth and CO_2_ removal efficiency increased with increasing CO_2_ concentration. The lipid content of the algae also increased with time and the maximum lipid content observed was 10%. Based on 18S rDNA technique, the halotolerant algae was identified as *Chlorella* sp.

## Competing interests

All authors declare that they have no competing interest.

## Authors’ contributions

UR contributed in growth measurements, CO2 removal, alkalinity, pH variation studies. BB helps in algal DNA isolation, PCR amplification, and dendogram preparation. SS is overall guide for the R & D work. All the authors read and approved the final manuscript.
